# Effects of condensed tannins on behavior and performance of a specialist aphid on aspen

**DOI:** 10.1002/ece3.9229

**Published:** 2022-08-23

**Authors:** Bárbara Díez Rodríguez, Karen J. Kloth, Benedicte Riber Albrectsen

**Affiliations:** ^1^ Department of Plant Ecology and Geobotany Philipps‐University Marburg Marburg Germany; ^2^ Department of Plant Physiology Umeå Plant Science Centre Umeå Sweden; ^3^ Laboratory of Entomology Wageningen University and Research Wageningen The Netherlands

**Keywords:** *Chaitophorus tremulae*, condensed tannins, electric penetration graph (EPG), *Populus tremula*, xylem feeding

## Abstract

Genes involved in plant defences against herbivores and pathogens are often highly polymorphic. This is a putative sign that balancing selection may have operated reciprocally on the hosts and their herbivores. Spatial and temporal variations (for example, in soil nutrients and the plants' ontogenetic development) may also modulate resistance traits, and thus selection pressures, but have been largely overlooked in theories of plant defences. Important elements of defences in *Populus tremula* (hereafter aspen) are phenolic compounds, including condensed tannins (CTs). Concentrations of CTs vary considerably with both variations in external factors and time, but they are also believed to provide genotype‐dependent resistance, mainly against chewing herbivores and pathogens. However, evidence of their contributions to resistance is sparse. Detailed studies of co‐evolved plant–herbivore associations could provide valuable insights into these contributions. Therefore, we examined correlations between CT levels in aspen leaves and both the feeding behavior and reproduction of the specialist aspen leaf aphid (*Chaitophorus tremulae*) in varied conditions. We found that xylem sap intake and probing difficulties were higher on genotypes with high‐CT concentrations. However, aphids engaged in more nonprobing activities on low‐CT genotypes, indicating that CTs were not the only defence traits involved. Thus, high‐CT genotypes were not necessarily more resistant than low‐CT genotypes, but aphid reproduction was generally negatively correlated with local CT accumulation. Genotype‐specific resistance ranking also depended on the experimental conditions. These results support the hypothesis that growth conditions may affect selection pressures mediated by aphids in accordance with balancing selection theory.

## Graphical abstract

The contributions of condensed tannins (CTs) to aspen resistance provided clear indications of complex interactions between host genotype and both environmental and ontogenetic factors. Induced foliar CT levels correlated negatively with aphid reproduction, and aphids on high‐CT genotypes spent more time on probing and xylem feeding and they more frequently showed signs of penetration difficulties. However, genotype‐resistance rankings depended on both plant age and growth conditions.

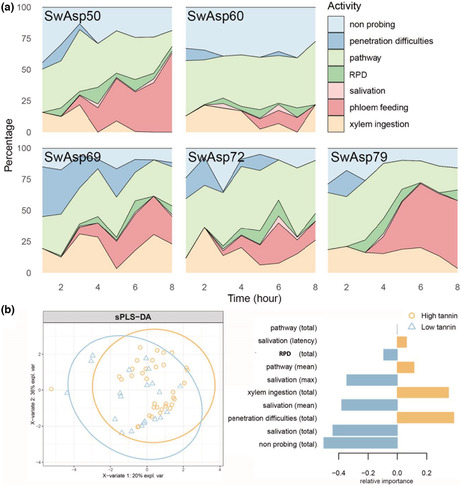

## 1 INTRODUCTION

Compounds involved in plants' defences against herbivores and pathogens have well‐established variations with ontogenetic factors (Cope et al., 2021) and soil nutrients (Decker et al., 2017). Thus, according to recent plant defence theories, potential effects of such factors on plants' resistance must be considered in efforts to elucidate evolutionary interactions between plants and consumers (Barton & Boege, 2017; Hahn et al., 2019). For example, López‐Goldar et al. (2020) found that investments in both growth and defence traits of pine trees were high in rich environments, leading to positive correlations between growth and resistance, but negative correlations were detected in poorer environments, where constitutive defences were prioritized over inducible defences. Thus, variations in resistance could be shaped by growth conditions and contribute to the diversity of defence traits in natural tree populations.

Aspens are deciduous, early succession trees that are widely distributed in forests of the Northern hemisphere (Rogers et al., 2019). Aspen trees have also been used as models in studies of intraspecific differences in defence chemistry (Häikiö et al., 2009; Hartikainen et al., 2009; Keefover‐Ring et al., 2014; Lindroth & Hwang, 1996; Rubert‐Nason & Lindroth, 2021), inter‐ and intraspecific variation in defence genes (Bernhardsson et al., 2013; Wang et al., 2016a, 2016b, 2020), environmental modulation of metabolic priorities (Bandau et al., 2015, 2021; Cole et al., 2021; Cope et al., 2021; Decker et al., 2017; Dettlaff et al., 2018; Gaur et al., 2022), and molecular mechanisms involved in induced defence responses (Barbehenn & Peter Constabel, 2011; Barrios‐San Martín et al., 2014; Ramírez et al., 2004; Ullah et al., 2018, 2019). They are also regarded as strong foundation species in temperate and boreal forests (Cole et al., 2021), hosting genotype‐specific communities of arthropods (Barker et al., 2019; Robinson et al., 2012), and pathogens (Albrectsen et al., 2010; Bandau et al., 2015; von Bargen et al., 2020).

As summarized by Lindroth and St. Clair (2013), aspen trees have three basal strategies for resisting and avoiding damage by herbivores and other threats: tolerance, escape (by fast growth), and defence (by C‐based, mainly phenolic, chemicals). Aspen defence phenolics include polymeric condensed tannins (CTs) and salicinoid phenolic glycosides (SPGs), collectively known as total phenolics (TPs) that may account for more than 25% of a leaf's dry weight (Lindroth & Hwang, 1996). Aspen genotypes can be classified in terms of their relative investment in CTs as high or low‐CT genotypes (Bandau et al., 2021). However, tannin concentrations also vary with tissue, age, soil nutrients, and ontogenetic factors (Häikiö et al., 2009, Decker et al., 2017; Cole et al., 2021). CTs in Populus spp. have mainly been associated with defences against microorganisms, such as endophytic fungi (Bailey et al., 2005) and fungal pathogens (Bandau et al., 2021; Ullah et al., 2018, 2019), but studies also suggest they impair arthropod chewing performance (Barbehenn & Peter Constabel, 2011; Rubert‐Nason & Lindroth, 2021).

Aspen canopies host mainly specialist arthropod herbivores, with substantial genotype‐dependent variations in their community composition (Barker et al., 2019; Robinson et al., 2012, 2016). *Chaitophorus* aphids include circa 100 species on salicaceous tree species of which several specialists on aspen that appears to select some host genotypes over others (Barker et al., 2019). In hybrid poplar plantations, *Chaitophorus* can also reportedly reach pest status on some taxa (Ramírez et al., 2004; Yali et al., 2010). Thus, analysis of *Chaitophorus* aphids' choices of host genotypes could provide valuable insights into defence traits that influence their host preferences and associated selection pressures.

Aphids have piercing‐sucking mouthparts (stylets) that enable a highly specialized feeding mode (Tjallingii & Hogen Esch, 1993), suggesting tight co‐evolution between host and aphid (Dixon, 1998). Before reaching a vascular bundle and during passive phloem sap ingestion, an aphid must overcome several plant defences for example via the secretion of saliva with effectors derived from the aphid itself or its endosymbionts (e.g., Züst & Agrawal, 2016). Probing activities also induce biosynthesis of specialized metabolites, such as salicylic acid, indole glucosinolates, and camalexin in *Arabidopsis* (Kettles et al., 2013; Kim et al., 2008), and phenolic compounds in *Vaccinium* (Ranger et al., 2007). While aphids commonly feed on phloem, they also occasionally ingest xylem sap (Daniels et al., 2009; Pompon et al., 2011; Spiller et al., 1990). Although CT levels could explain the differences in colonization of aphids on some poplar genotypes (Ranger et al., 2007), and CTs putatively reduce the fecundity of aphids on aspen (Gaur et al., 2022), we have little detailed knowledge of the mechanisms involved in CTs' contributions to plants' resistance or the behavioral patterns they might affect.

We used the electrical penetration graph (EPG) recording technique to study the feeding behavior of the aspen leaf aphid (*Chaitophorus tremulae*) on aspen (*P. tremula*). We hypothesized that aphids express varied feeding difficulties depending on aspen genotype, and that genotype‐specific feeding barriers correlate with aphid reproduction rate on the same genotypes in the same environment. We then asked if resistance traits were constant across experimental conditions by comparing the resistance rank of the same genotypes in different environments. Finally, we used CT ranking of genotypes to suggest candidate aphid resistance traits linked to leaf CTs.

## 2 MATERIAL AND METHODS

### 2.1 Biological material

#### 2.1.1 Plants

The Swedish aspen (SwAsp) collection is a library of circa 100 *P. tremula* genotypes with substantial variation in growth traits, as described by Luquez et al. (2009). The library is kept in tissue culture at the Umeå Plant Science Centre (UPSC) for propagation purposes, and for two decades, genotypes from the collection have been grown in up to four common gardens that allow studies of genotype and environmental effects. For the laboratory and greenhouse studies reported here, potted SwAsp plantlets were prepared from the UPSC tissue cultures by propagation (at ~20°C and ~60% RH, with 18/6‐h light/dark cycles). Plantlets (2–6 months old and up to 60‐cm tall) were used for the indoor studies. Field studies were performed with small (less than 450‐cm tall) 7‐year‐old trees in the TanAsp common garden where SwAsp genotypes with substantially varying leaf contents of CTs were planted in 2010 (Bandau et al., 2021). Genotypes with both low innate ability (SwAsp genotypes 50 and 60) and high innate ability (SwAsp genotypes 65, 69, 72, and 79) to produce and store CTs were used in the studies, as detailed in the Appendix, Figure S1.

We selected two “high‐CT” genotypes (SwAsp65 and 72) and two “low‐CT” genotypes (SwAsp50 and 60) for use in a field trial (described below), but SwAsp65 was not available for propagation at the time of the indoor experiments, so it was replaced by two high‐CT genotypes that propagated equally well (SwAsp69 and 79).

#### 2.1.2 Aphid culture

Free‐living specialist *Chaitophorus* aphids are commonly found on aspen across Sweden, as demonstrated by records compiled in the Swedish species database Dyntaxa (Taxon ID: 245999; Liljeblad, 2021). In late July 2016, circa 30 *Chaitophorus* individuals were collected from 40‐year‐old *P. tremula x tremuloides* hybrids at Skogsforsk research station in Sävar, Sweden, next to a SwAsp garden. As several aphid species may live together in single colonies on aspen (Raizada et al., 2021), we placed aphids singly to reproduce on a set of *P. tremula x P. tremuloides* T89 hosts in the propagation facility to avoid conditioning to a particular SwAsp genotype. Each colony was allowed to develop for 16 days, and the colony that reproduced most strongly was selected to culture for the studies reported here. To prevent the development of oviparous females, the culture was maintained under long‐day conditions (~20°C, with 18/6‐h light/dark cycles). The aphids were preliminarily identified as “the aspen leaf aphid” (*Chaitophorus tremulae*) according to Blackman and Eastop (1994) and Stroyan (1977). The species' identity was later confirmed by Drs B. Dransfield and B. Brightwell by sharing photographic material (Appendix, Figure S2) through services provided by Influential Points (https://influentialpoints.com; Influentialpoints, 2021).

### 2.2 Bioassays

#### 2.2.1 Aphid probing behavior

To study aphid feeding behavior, we used an EPG system (ten Broeke et al., 2013). In this technique, originally developed by Mclean and Kinsey (1967), an electrical circuit is established with an aphid acting as “bio‐electrode.” The insect's probing activities are captured as electrical patterns that can later be translated into detailed feeding behaviors (Backus et al., 2019; Tjallingii, 1988). The method is well‐established for recording hemipteran feeding behavior, including nonprobing periods, pathway phases, cell penetrations, penetration difficulties, salivation in the phloem, phloem feeding, and xylem sap drinking. The aspen plants were sufficiently small (10‐ to 20‐cm tall) to include aphids on five plants (and thus conduct five parallel aphid probing tests) in each 8‐h EPG recording session. In total, we performed at least 10 replicated aphid probing trials per genotype. One plantlet of each of the five SwAsp genotypes used in the indoor studies was randomly selected for each recording (Appendix, Figure S3). An aphid was placed on the adaxial side of a mature fully expanded leaf (number 7–10 from the top) of each of these plants with a 1‐ to 2‐cm‐long, 18‐μm‐diameter gold wire glued to its dorsum. The other end of the wire was connected to a Giga 8d DC amplifier (EPG systems, 2021), and linked to a computer to capture and store the EPG‐generated waveforms for later interpretation (Kloth et al., 2021; ten Broeke et al., 2013). Stylet+d and Stylet+a software packages provided by EPG systems (2021) were used to record and annotate the EPG waveforms (Appendix, Figure S3). Behavioral variables, listed in Table 1, were calculated using R v. 4.0.4 software (R Core Team, 2020), and the procedure presented by Kloth et al. (2021), except that waveforms that did not occur were assigned zero values for duration measurements, missing values for calculating mean values, and total durations for latency times (i.e., the time from the start of a recording until the start of another behavior). Behaviors that were ongoing at the end of a recording session (after 8 h) were included in their short, truncated form.

**TABLE 1:** Aphid probing behavior parameters on each of five aspen genotypes derived from 8‐h electrical penetration graph (EPG) recordings (means ± SE)

Behavior	SwAsp50 *n* = 10	SwAsp60 *n* = 11	SwAsp69 *n* = 11	SwAsp72 *n* = 13	SwAsp79 *n* = 12
Total duration no probing	119.7 ± 28.3ab	172.5 ± 34.1b	54.1 ± 12.6a	65.4 ± 13.7a	86.5 ± 29.2ab
Pathway events (no. C/aphid)	16.1 ± 1.8b	16.9 ± 1.7b	11.3 ± 1.3a	15.4 ± 2.5ab	15.2 ± 2.7ab
# C < 3 min	14.7 ± 1.8b	14.5 ± 1.6b	8.8 ± 1.2a	13.5 ± 2.3ab	12.2 ± 2.5ab
Mean duration C	9.8 ± 1.4	10.9 ± 1.2	11.8 ± 1.4	14.4 ± 1.9	10.1 ± 1.1
Total duration C	146.1 ± 19.4ab	168.7 ± 14.2b	125.9 ± 19.5a	181.8 ± 19.7ab	140.6 ± 21.2ab
PD rate (# PD/min C)	0.6 ± 0.1	0.6 ± 0	0.6 ± 0.1	0.6 ± 0.1	0.5 ± 0
Repetitive potential drops (no. RPD/aphid)	2.4 ± 0.6b	2.4 ± 1.1ab	2.1 ± 0.9ab	2.4 ± 1ab	0.9 ± 0.2a
Mean duration RPD	15.5 ± 5.6	7.3 ± 3.1	11.5 ± 5.2	17.5 ± 5.6	30.8 ± 9.2
Total duration RPD	29.2 ± 7.4b	14 ± 6.4ab	13.8 ± 6.2a	27.4 ± 10.4ab	28.6 ± 9.2ab
Salivation events (# E1/aphid)	1.2 ± 0.3	0.6 ± 0.4	0.5 ± 0.3	0.8 ± 0.3	0.6 ± 0.2
Mean duration E1	7.6 ± 2.6	6 ± 3.1	5.2 ± 0.5	8.4 ± 3.5	4.5 ± 0.9
Total duration E1	11.6 ± 6.7b	6 ± 5.1a	2.6 ± 1.2ab	4.7 ± 2.1ab	2.5 ± 0.9ab
Max duration E1	9.3 ± 4.1	8.8 ± 5.7	6.2 ± 0.7	9.7 ± 3.3	4.8 ± 0.9
Latency to first E1	295.4 ± 41.4	397.4 ± 40.5	401.1 ± 36.1	376.5 ± 35.3	362.5 ± 36.7
No. of E1 not followed by E2	0.4 ± 0.2	0.1 ± 0.1	0.1 ± 0.1	0.2 ± 0.1	0 ± 0
% E1 in total phloem phase (E1 + E2)	8.2 ± 4.5	17 ± 9.9	6.8 ± 2.9	3.9 ± 1.9	2.2 ± 0.4
Phloem ingestion events (no. E2/aphid)	0.8 ± 0.2	0.4 ± 0.2	0.4 ± 0.2	0.5 ± 0.2	0.6 ± 0.2
% Aphids performing E2	70%	27%	36%	31%	50%
Mean duration E2	138.7 ± 47.1	64.7 ± 45	172.4 ± 65.7	130.2 ± 35	208.1 ± 24
Total duration E2	102 ± 38.2b	20.9 ± 14.7a	62.7 ± 34ab	50.4 ± 23.4ab	115 ± 35.9ab
Total duration of E2 events >10 min	102 ± 38.2b	20.3 ± 14.7a	62.7 ± 34ab	50 ± 23.2ab	115 ± 35.9ab
Max duration E2	142 ± 46.2ab	76.3 ± 42.7a	172.4 ± 65.7ab	149.2 ± 28ab	218.8 ± 19b
Penetration difficulties (no. F/aphid)	0.2 ± 0.1a	0.6 ± 0.3ab	1.3 ± 0.3b	0.8 ± 0.3ab	0.5 ± 0.2a
% Aphids performing F	20%a	45%ab	82%b	38%ab	33%ab
Total duration F	12.4 ± 10.1a	20.2 ± 9.4a	103.4 ± 30.4b	42.9 ± 25.4a	20.7 ± 11a
Xylem ingestion events (no. G/aphid)	1.6 ± 0.4	1.7 ± 0.3	1.7 ± 0.3	2.1 ± 0.3	1.8 ± 0.3
% Aphids performing G	70%	100%	91%	100%	100%

*Note*: Different letters indicate significant (*p* < .05) genotype differences (according to the Mann–Whitney U test). Probing behavior nomenclature follows Tjallingii and Hogen Esch (1993).

Abbreviations: C, pathway; E1, salivation in phloem; E2, phloem ingestion; F, penetration difficulties in cell wall; G, xylem ingestion; PD, potential drop; RPD, repetitive PD.

#### 2.2.2 Reproduction studies

Aphid reproduction was assessed in the greenhouse with plantlets identical to those used for the EPG laboratory studies. The same genotypes (7‐year‐old plants) were also assessed in the field except that (as already mentioned) genotype SwAsp65 used in the field was unavailable for propagation during 2016–2017, so it was replaced in laboratory and greenhouse experiments by two other high‐CT genotypes (SwAsp69 and SwAsp79).

**In the greenhouse.** Experiments in this setting were conducted during three periods: Jan.19 to Feb. 20 (*n* = 17), Feb.10 to March 25 (*n* = 16), and Oct. 27 to Nov.16 (*n* = 72). The genotypes used were (with sample sizes in the mentioned order of experiments in parentheses): SwAsp50 (*n* = 4, 6, 14), SwAsp60 (*n* = 6, 3, 11), SwAsp69 (*n* = 2, 2, 14), SwAsp72 (*n* = 2, 3, 14), and SwAsp79 (*n* = 3, 4, 14). To avoid aphid escape during these experiments, plantlets (~20‐ to 25‐cm tall) were placed in 120 × 55‐cm trays, with a few millimeters of standing water in the bottom, a drop of detergent added to break the surface tension, and at least 5 cm between leaves of neighboring plants. The trays were placed on tables that enabled close inspection of each plant. In the third experiment, slightly larger plantlets (20–60 cm) were placed directly on the floor in individual saucers, with sufficient spacing to allow watering and examination of reproduction parameters on three occasions without moving the plants.

To infest a plant, a single nymph, less than 24 h old, was placed with a fine paintbrush on the first expanded mature leaf closest to the top of the plant. During the first two greenhouse experiments, the time to first reproduction (prereproductive time) was recorded, then newborn nymphs were counted and removed daily until the founder stopped producing nymphs. In the last experiment, as more experimental plants were used, nymph production was counted without removal 1, 7, 13, and 22 days postinfestation (dpi), distinguishing between adults and nymphs. If winged aphids had developed on any plant the experiment had been terminated.

**In the field.** Aphid reproduction was assessed, in the TanAsp common garden in Vindeln (Bandau et al., 2021), on 44 small trees of four genotypes: SwAsp50 (*n* = 11), SwAsp60 (*n* = 12), SwAsp65 (*n* = 10), SwAsp72 (*n* = 11). Apterous viviparous adults from the culture in Umeå were placed in 0.5 ml Eppendorf tubes, and less than 12 h later transported for one hour in a cooler (Adriatic 24 L, on refreezable ice blocks precooled to −20°C) to the field site. In the field, the Eppendorf tubes were opened individually and placed in mesh bags, each enclosing a fully mature leaf per plant (Appendix, Figure S2), and left to reproduce. On September 2nd (17 dpi) aphids in the mesh bags were counted.

### 2.3 Leaf harvests and studies of condensed tannins

To assess leaf CT contents, a fully developed mature leaf was harvested with a sharp cut just under the leaf base, leaving most of the petiole on the plant. Leaves were placed in labeled plastic bags and flash‐frozen in liquid nitrogen (in the laboratory or greenhouse) or placed on dry ice (in the field). Samples were placed in a −20°C freezer (immediately if harvested indoors, or after transport for circa an hour to Umeå in the cooler on dry ice if harvested in the field). Leaf samples were lyophilised in Umeå using a Labogene 3450 system, and ground to a fine powder with a mortar and pestle for chemical analyses.

CT induction by the aphids was assessed using a balanced experimental design with four biological replicates per genotype (Appendix, Figure S1). Offspring were counted at 17 dpi, aphids were removed, and two leaves were harvested per plantlet: one low on the plant as “constitutive,” assuming that induction of CTs is negligible in old leaves (Papazian et al., 2019), and the leaf hosting the aphid colony as a proxy of local induction potential.

In addition, to monitor seasonal changes in CT levels in the field, leaves were collected repeatedly from each experimental tree on July 13, Aug. 15, and Sept. 2, 2016. As the same plants were used for the field reproduction study, to minimize potential effects of induction caused by the treatment, leaves were harvested from a different branch of the canopy than the one holding the mesh bag with aphids.

Foliar CTs were quantified using the acid‐butanol assay protocol of Porter et al. (1985) as described by Bandau et al. (2021). Briefly, 10 mg portions of leaf powder were used to quantify the contents in mg/g dry weight, and photometric measurements were acquired using multiwell plates and a SpectraMax 190 Microplate Reader (Molecular Devices Corporation, Sunnyvale, California).

### 2.4 Algorithms and statistical analyses

Behavioral variables from the EPG recordings (Table 1) and life‐table parameters (total numbers of aphids, prereproductive period, and the intrinsic rate of increase) were analysed with one‐way ANOVA models for normally distributed data, and groupwise Kruskal–Wallis tests or pairwise Mann–Whitney U tests for nonparametric data. The normality of model residuals was tested with the Shapiro–Wilk test.

Aphid reproductive parameters were calculated as follows: intrinsic growth (*r*_m_) = 0.738 (log_e_ *M*_*d*_)/*d*, where *d* is the prereproductive period in days, *M*_*d*_ is the total number of nymphs, and 0.738 is a correcting constant estimated for several aphid species by Wyatt and White (1977). The aphid population doubling time (DT) = log_e_/*r*_m_.

For the experiment in which nymph reproduction was measured repeatedly, the significance of genotype and age effects on aphid reproduction was tested with a two‐way repeated measures ANOVA.

Reproduction in field and greenhouse conditions could not be compared using a single model due to the overdispersion of data. Instead, the effects of genotype and age on reproduction in the two settings were tested separately. As the aphid reproduction data were counted, a negative binomial distribution model was chosen, as implemented in the lme4 package (Bates et al., 2015), and the check_overdispersion function (*performance* R package) was used to confirm the absence of overdispersion.

In accordance with Bandau et al. (2021), to explore the potential effects of CTs on aphid probing behavior, we divided SwAsp genotypes into high‐CT (SwAsp69, 72, and 79) and low‐CT producers (SwAsp50 and 60). Sixteen EPG variables were subjected to sparse partial least‐squares analyses (sPLSDA). These included total durations of nonprobing, pathway, salivation, phloem feeding, sustained phloem feeding (>10 min), penetration difficulties, xylem sap drinking, and repetitive potential drops (RPDs). Four were mean durations (of pathway, salivation, phloem feeding, and RPDs). The others were the rate of potential drops, maximum durations of salivation and phloem feeding, and latency to salivation. However, the sPLSDA did not include counts (frequencies of events) or proportions (percentages of aphids or proportions of time). The R package MixOmics (Rohart et al., 2017) was used for the sPLSDA test, and to minimize the classification error rate, the number of components was set to two, each with a minimum of 10 variables to use.

Statistical analyses were conducted using R version 4.0.4 (R core team, 2020), and scripts are available at https://doi.org/10.5281/zenodo.5578109 under “models.R" and “READ ME.txt.”

## 3 RESULTS

### 3.1 Aphid probing varied with aspen genotype

**FIGURE 1:**
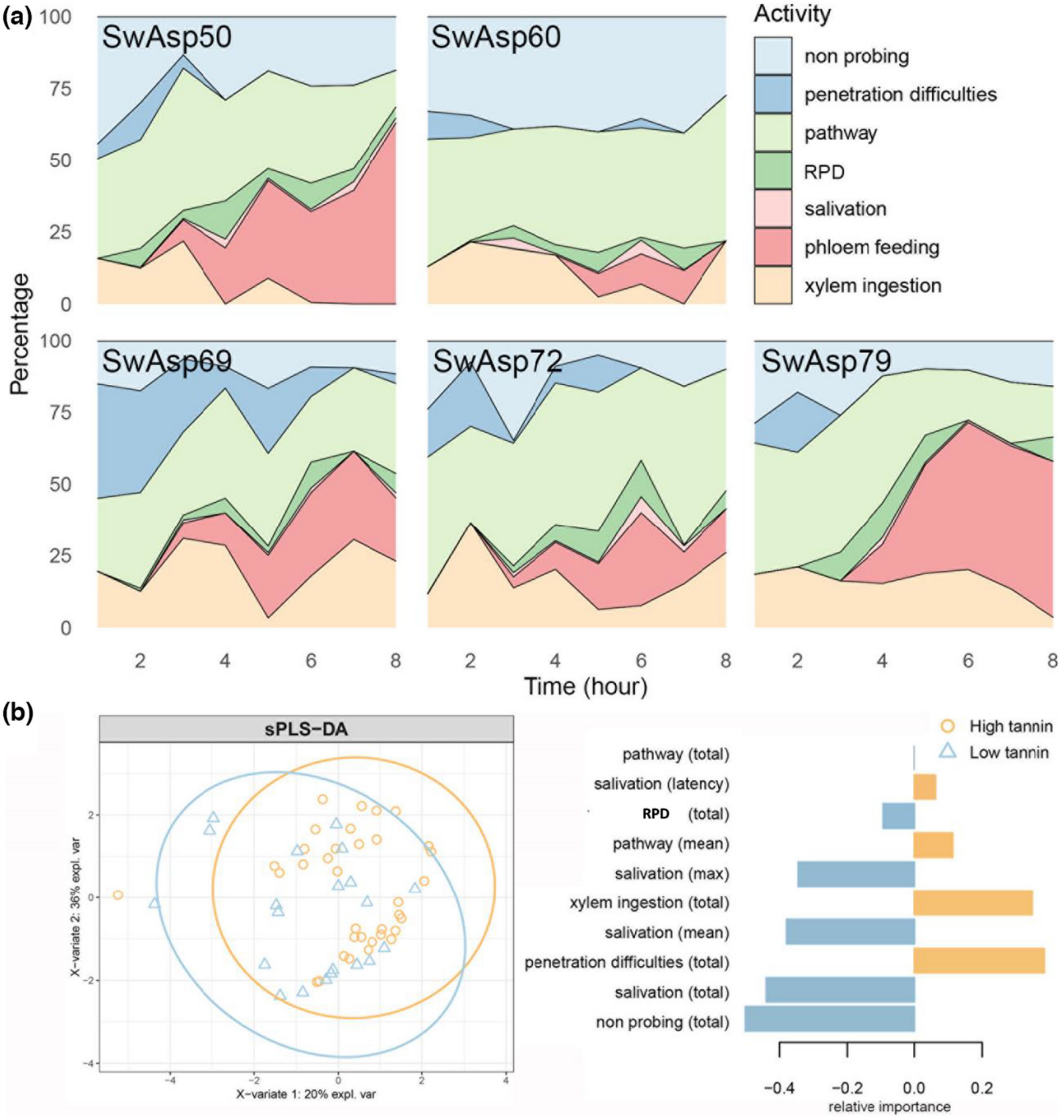
Activity diagram of feeding behavior of the aspen leaf aphid (*Chaitophorous tremulae*) on five aspen genotypes during 8‐h electrical penetration graph (EPG) recordings. (a) Percentage of time that aphids spent on activities organized after SwAsp genotype. Pathway = stylet movements towards the vascular bundle; RPD = repetitive potential drops; salivation = salivation in the phloem. (b) Feeding activities that differed between aphids on SwAsp genotypes according to their innate ability to produce and store condensed tannins (CTs). Activities that significantly differed (*p* < .05) included nonprobing and salivation, which lasted longer on low‐condensed tannin (CT) genotypes (SwAsp50 and 60), while aphids on high‐CT genotypes (SwAsp69, 72, and 79) spent more time on xylem feeding and were generally more active recorded activities of 10–13 aphids on each aspen genotype, were used in the comparison.

EPG‐based analysis of aphid probing behavior on *P. tremula* plantlets revealed significant between‐genotype differences (Table 1, Figure 1). Most prominently, on SwAsp50 and 79 at least 50% of the aphids reached phloem feeding (E2), while on SwAsp60, 69, and 72 only 27%–36% of the aphids reached this phase and spent more time on prephloem feeding activities (nonprobing, pathway, or penetration difficulties). The total time spent on salivation (Total duration E1), which prepares the aphid for phloem feeding, was higher on SwAsp50 than on SwAsp79. The total duration of inactivity (no probing) was longer on SwAsp60, whereas the “pathway” phase (Total duration C), which involves the prephloem stage, was longer on SwAsp72. The total time spent on C was lowest on SwAsp69, and aphids also showed most signs of penetration difficulties (F), which often occurred at the beginning of recording periods, on this genotype (Figure 1a). Repetitive potential drops (RPDs), observed on all genotypes, indicate recurrent puncturing of a cell membrane with subsequent intracellular activities before initiation of phloem feeding (Tjallingii et al., 2010; Tjallingii & Gabryś, 1999). On SwAsp79, two‐thirds of the RPD behaviors progressed to phloem sap ingestion (0.6 E2 out of 0.9 RPD events per aphid), compared with less than 50% on the other genotypes. Surprisingly, almost all aphids spent a substantial amount of time on xylem sap drinking (G), amounting to 10%–20% of the eight‐hour recording durations (Table 1).

### 3.2 Probing correlated with reproduction under same conditions

**FIGURE 2:**
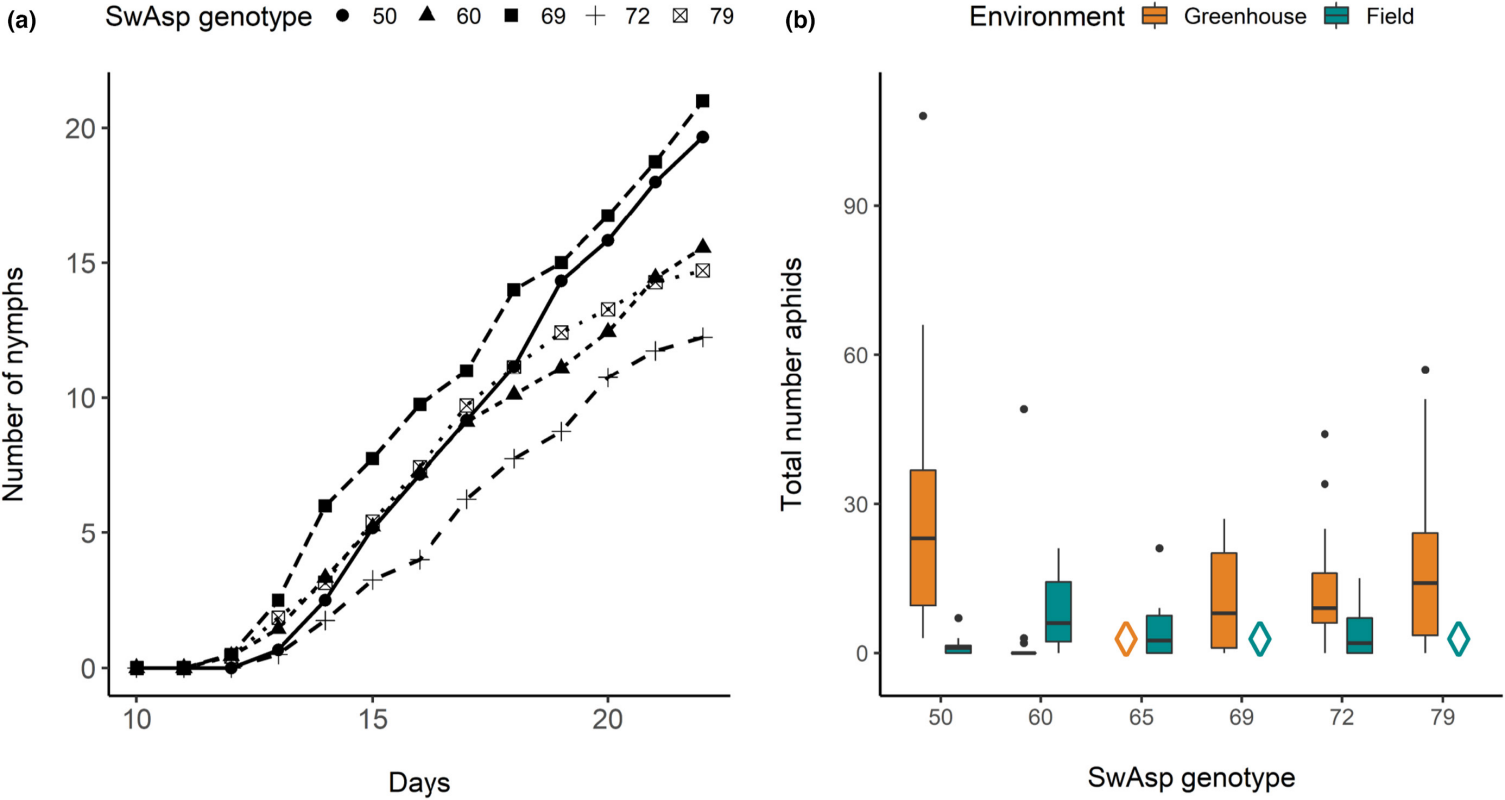
Aphid population development on aspen genotypes differed in two experimental settings: greenhouse and field (Table 4). (a) Aphid growth curves based on daily counts of newborn nymphs from one founder individual in a greenhouse experiment with low‐CT genotypes SwAsp50 and 60 plus high‐CT genotypes SwAsp69, 72, and 79. Sample sizes: *n* ≥ 11 per SwAsp genotype (details in Table 1). (b) Final population sizes at 17 days postinfestation in the greenhouse (orange) and field (green). Horizontal line inside the boxes = medians, boxes contain 50% of data, whiskers 95% quantiles, and points indicate outliers. SwAsp genotypes that differed in the two experimental settings are indicated with diamonds, and (as above) orange and green indicate observations in the greenhouse and field settings, respectively.

In greenhouse experimental setups, the reproductive effort was assessed in terms of cumulative numbers of newborn nymphs per adult (Figure 2). The resulting genotype‐specific reproduction curves significantly differed (Figure 2a, repeated measures ANOVA p = 0.001). Reproduction was fastest on SwAsp69 and 50, and the high rate of successful phloem feeding on Swasp50 (indicated by the EPG recordings, Table 1) suggests that SwAsp50 was the more susceptible of the tested genotypes. Surprisingly, despite indications in EPG analyses of probing difficulties on SwAsp69, the final aphid population numbers on this genotype did not significantly differ from those on SwAsp50. A circa three days shorter prereproductive period (d, Table 2) gave aphids on SwAsp69 a reproductive head start compared with aphids on SwAsp50, which could explain why the final population sizes were similar on the two genotypes. Population growth parameters of aphids on genotype SwAsp79, which reached the phloem feeding phase in 50% of the EPG recordings (Table 1), resembled those of aphids on genotype SwAsp50. However, due to an extended prereproductive period (Table 2), the final population size on SwAsp79 did not differ significantly from the population size on genotype SwAsp60, on which aphids spent long periods of nonprobing activities during the EPG recordings (Table 1). A long prereproductive period (*d*), low reproduction rate (*r*_m_), and high doubling time (DT) were recorded for aphids on SwAsp72. Accordingly, aphid fecundity was lowest on this genotype (Figure 2, Table 2), and generally, SwAsp72 was also ranked as the most resistant genotype. However, between‐genotype differences were not strongly supported by post hoc comparisons of single traits except for “probing activity level,” which was high on SwAsp72. In conclusion, combinations of feeding traits were needed to explain genotype susceptibility and resistance differences, although aphids on high‐CT genotypes commonly spent high proportions of time on probing activity, which appeared to correlate with low fitness parameters.

**TABLE 2:** Life table parameters of specialist aspen leaf aphids (*Chaitophorous tremulae*) on a set of European aspen (*Populus tremula*) genotypes from the Swedish Aspen collection in replicated greenhouse experiments (Appendix, Figure S1).

Genotype	*d*	*r*_m_	DT	*r*_m_	DT
	1st replicate			2nd replicate	
SwAsp50	14.2 ± 1.2	0.18 ± 0.01	3.91 ± 0.28	0.13 ± 0.02	7.12 ± 1.36
SwAsp60	12.9 ± 0.9	0.18 ± 0.01	3.96 ± 0.13	0.10 ± 0.07	30.91 ± 22.6
SwAsp69	11.7 ± 0.3	0.17 ± 0.05	6.79 ± 9.61	0.14 ± 0.03	4.67 ± 0.48
SwAsp72	14.0 ± 1.5	0.15 ± 0.03	5.26 ± 2.22	0.10 ± 0.02	11.10 ± 4.04
SwAsp79	13.4 ± 0.8	0.16 ± 0.01	4.45 ± 9.38	0.16 ± 0.01	4.59 ± 0.45

*Note*: Equations indicated in the text. Values in mean ± SE.

Abbreviations: *d*, Prereproductive period (from newborn nymph to first reproduction in days); DT, population doubling time; *r*_m_, Intrinsic rate of increase.

### 3.3 Genotypic reproductive rankings depended on experimental conditions

Comparisons of aphid population development on 7‐year‐old trees in the field and young plantlets in the greenhouse clearly suggested that resistance is influenced not only by genotype but also by experimental conditions. This conclusion was supported by the significant effects of the experimental condition and its interaction with genotype on aphid population development (Table 3). In addition, SwAsp50 was the most susceptible genotype in the greenhouse and most resistant in the field, while SwAsp60 was most resistant in the greenhouse and most susceptible in the field (Figure 2b). By contrast, hardly any extreme values of any EPG‐trait were recorded on high‐CT genotypes SwAsp72 and 79, and their relative resistance levels (measured in terms of both EPG and reproductive performance traits of the aphids) varied least between experimental situations.

**TABLE 3:** Summary of results of 2‐way ANOVA of effects of aspen (SwAsp) genotype, experimental condition (EC), and their interaction (SwAsp:EC) on final aphid population sizes (as shown in Figure 2b).

	df	Sum Sq	Mean Sq	*F* value	*pr*(>*F*)
SwAsp	5	4305	861.0	2.961	.01578*
EC	1	2454	2453.8	8.440	.00457**
SwAsp:EC	2	3564	1782.0	6.129	.00314**
Residuals	95	27,619	290.7		

Note

*p < .05

**p < .01.

### 3.4 Indications that CTs may restrict probing and reproduction

**FIGURE 3:**
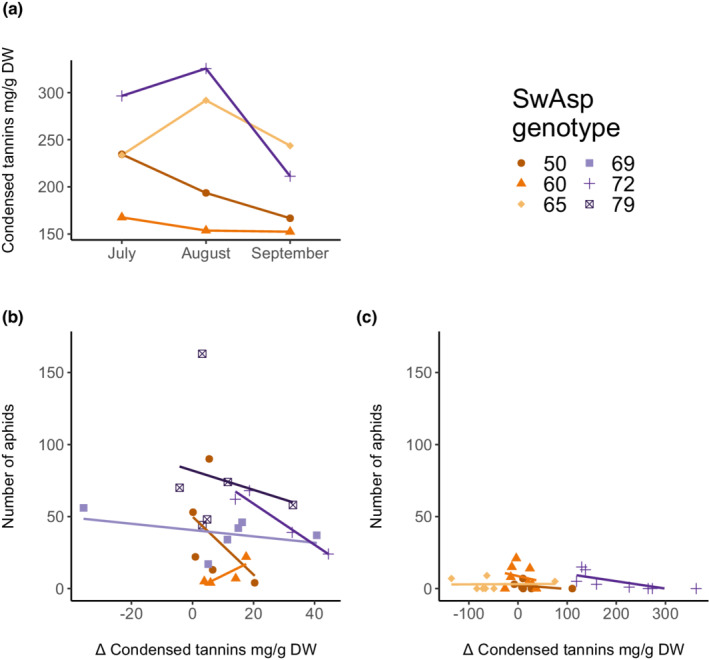
Seasonal plasticity of foliar concentrations of condensed tannins (CT) and relationships between condensed tannin induction and aphid population development: (a) Seasonal change in CT contents in 7‐year‐old trees in the TanAsp common garden; *n* = 10–12 per genotype. Relationship between aphid population development and Δ condensed tannins (the difference between constitutive and locally induced CT values of a plant, in mg/g DW) as assessed in the greenhouse (b) and in the field (c); for greenhouse values also see Appendix S5B and S5C. A tendency to a negative relationship was not statistically supported, see Table 4. Aphid population sizes were measured 17 days postinfestation in both (b) and (c).

To study the relationship between aphid fitness and foliar tannin concentrations, tannin levels were measured under both field and greenhouse conditions. Tannin concentrations in aspens in the field were measured on three occasions during the growing season. The results showed that levels differed, as expected, among genotypes (Figure 3a, ANOVA: *F*_3,20_ = 13.63, *p* < .0001) and varied insignificantly with time (ANOVA: *F*_2,20_ = 1.567, *p* = .23). Weak negative correlations between foliar CTs and aphid population sizes were not supported statistically (GLMM: *p* = .45 and *p* = .31 in field and greenhouse settings, respectively; Figure 3b,c; Table 4). Interestingly, in the induction experiment, relationships between leaf CTs and aphid reproduction appeared to depend on leaf type, with aphid reproduction correlating negatively with induced tannin levels, and aphid reproduction correlating positively with constitutive CT levels (Appendix, Figure S5B and S5C, Table S6). In conclusion, we confirmed there were substantial genotypic‐dependent changes in leaf CT contents in response to aphid infestation, particularly in high‐TC genotypes, and our results suggest that CT levels mostly correlated negatively (to varying degrees) with aphid fitness indicators.

**TABLE 4:** Effects on aphid reproduction of foliar‐induced tannin concentrations, with adjustment for host genotype. Summaries of GLMM tests with induced tannin concentration in the host (mg/g) as a fixed effect and host genotype (GT) as a random effect

Fixed effects	Estimate	SE	*Z*‐value	*p*‐value
Greenhouse				
Intercept	3.674	0.314	11.711	<.001***
Induced tannins	−0.009	0.009	−1.006	.314
Field				
Intercept	1.589	0.451	3.518	<.001***
Induced tannins	−0.003	0.004	−0.752	.451

*Note*: Because aphid fecundities are count data, we used a negative binomial distribution, and to avoid residual overdispersion, the greenhouse (Figure 3b) and field (Figure 3c) data were separately modeled. Genotype (random effect) added the following variances to the test intercepts: 0.35 (Greenhouse, obs = 25, GT = 5) and 0.001 (Field, obs = 31, GT = 4).

The significance level is ***p<.0001.

To assess tannin contents' effects on aspen leaf aphids' feeding behavior, the significance of differences in aphids' PG activities on low‐CT genotypes (SwAsp50 and 60) and high‐CT genotypes (SwAsp69, 72, and 79) was tested with sPLSDA. On high‐CT aspens, aphids experienced more penetration difficulties and engaged more in xylem sap ingestion than aphids on low‐CT hosts (Figure 1b). Aphids on low‐CT hosts engaged more in RPDs and salivation, indicating that they reached the phloem more often than on high‐CT hosts. Interestingly, however, this did not result in more phloem sap ingestion. Instead, aphids on low‐CT hosts engaged more in nonprobing activities, possibly because they were deterred by other defences in the phloem or upper cell layers or cuticle.

## 4 DISCUSSION

Our exploration of the contributions of condensed tannins (CTs) to aspen resistance against specialist aspen leaf aphids (*Chaitophorus tremulae*) through EPG‐based analyses of feeding behavior and reproductive success provided clear indications of complex interactions between host genotype and both environmental and ontogenetic factors. Induced foliar CT levels correlated negatively with aphid reproduction, and aphids on high‐CT genotypes spent more time on probing and xylem feeding and they more frequently showed signs of penetration difficulties. However, genotype resistance rankings depended on both plant age and growth conditions, and even aphids on a low‐CT genotype displayed genotype‐specific probing difficulties.

### 4.1 Genotype effects and their generality

Defence genes are highly polymorphic in aspen, a putative sign that individual trees and co‐evolved enemies are involved in reciprocal actions of selection (Wang et al., 2016a, 2016b). Our EPG monitoring of specialist *C. tremulae* aphids also indicated considerable differences in susceptibility and resistance among *P. tremula* genotypes. Genotypic variations were found in prolonged nonprobing periods, short phloem feeding activities, and xylem sap feeding, which are all indicators of antibiosis, as they coincide with components of low fitness, such as low fecundity and slow population development (Barrios‐San Martín et al., 2014; Table 1). On one of five genotypes (SwAsp50) aphids frequently reached the phloem feeding phase, which is a sign of susceptibility (ten Broeke et al., 2013). This was corroborated by high aphid population growth on this genotype in the greenhouse conditions, which resembled those of the EPG measurements. By contrast, we found extended periods with no probing on SwAsp60, and correspondingly poor reproduction under similar conditions, suggesting “antixenosis” (aka lack of host preference; Stout, 2013). The EPG suggested feeding barriers thus agreed with experienced aphid reproduction.

Aspen leaves are tough, dry, and rich in foliar CTs, but these features are more pronounced on trees in the field than on plantlets grown in the greenhouse. Accordingly, we found that resistance was generally higher in the field. However, the genotypes' resistance rankings also varied between indoor and outdoor experiments. Notably, SwAsp60 plantlets that had high resistance in the greenhouse appeared susceptible as young trees in the field. While these differences were found under varied experimental conditions with differences in both growth environment and plant developmental stages, the causes are unknown. However, similar differences in resistance associated with variations in growth environment have been detected in field studies of *C. leucomelas* aphids on poplar hybrids that had varied access to water (Ramírez & Verdugo, 2009), supporting the hypothesis that aspen resistance against *Chaitophorus* aphids may depend on the growth environment, as predicted by current plant defence theories (Barton & Boege, 2017; Hahn et al., 2019; López‐Goldar et al., 2020).

### 4.2 Tannin‐related defences

In the studies reported, here, negative relationships (within and among genotypes) between foliar tannin contents and aphid population development were consistently indicated. Tannins are located in the epidermal and upper mesophyll layers of leaves (Appendix, Figure S4; Barbehenn & Peter Constabel, 2011), which aphids encounter at the start of the stylets' pathway to a vascular bundle (Van Emden & Harrington, 2007). However, high tannin levels did not increase the number of short probes (<3 min) as expected. Instead, CT contents were positively related to the total time of probing (Figure 1b), a behavior that could raise risks of aphid‐vectored virus transmission (Perez et al., 1996). In addition, shorter salivation incidents and fewer RPDs were recorded on high‐CT aspens than on low‐CT aspens, suggesting more repeated puncturing of companion cells or sieve elements (Tjallingii et al., 2010; Tjallingii & Gabryś, 1999; Walker & Medina‐Ortega, 2012). These behavioral differences imply that there may be a negative relationship between CT contents and aphids' success in reaching the phloem (Figure 1b). Notably, we did not find that phloem sap ingestion itself was affected by CT content. Instead, increases in tannin contents were associated with increases in intake of xylem sap, a probing activity that has been explained by aphids' need to maintain the osmotic balance between their gut and hemolymph after phloem intake (Daniels et al., 2009; Pompon et al., 2011; Spiller et al., 1990). Therefore, the extent of xylem sap drinking we observed was exceptional, as most of the aphids engaged in the activity, and it continued for extended time periods (up to 20% of the recording time, Table 1). Although xylem sap contains some amino acids (Millard et al., 2006) and sugars (Secchi & Zwieniecki, 2011; Vogelmann et al., 1985), concentrations of nutrients in xylem are usually considered too low to meet aphids' energy needs (Novotny & Wilson, 1997). Thus, the association between xylem sap drinking and high‐CT profiles (Figure 1b) indicates that tannins may increase the need for osmoregulation in aphids, or the xylem sap intake may simply be an alternative to starvation.

Aphid population growth depends on numerous factors, including previous feeding history, host plant condition, and climatic variables (Campbell et al., 1974; Wyatt & Brown, 1977). During EPG recordings the aphids are restricted to feeding on a limited leaf surface during eight‐hour sessions. By contrast, fecundity assessments last longer and allow the aphids to build up local densities that may lead to crowding and induction differences. All these variables may potentially modify aphid feeding behavior, fitness, and hence herbivore pressure on the host. Interestingly, in a comparison of tree–herbivore networks of a northern and a southern common Swedish Aspen Garden, 1200 km apart (Luquez et al., 2009), it was suggested that the evolutionary impact on aspen–herbivore relationships is constrained by environmental factors (Robinson et al., 2016). In the northern garden, where the trees grow more slowly, leaf concentrations of phenolic defence compounds and the number of associated taxa (morphotypes) were higher than in the southern garden. Moreover, despite some overlap between the herbivore communities, the degree of modularity was higher in the north, indicating that genotype‐associated communities tended to be more defined in the harsher north, in accordance with predictions of how resource availability may shape selection pressures by altering defence phenotypes (Bakhtiari et al., 2019; López‐Goldar et al., 2020).

### 4.3 Standing genetic variations in aspen and aphids as selective forces

Many kinds of aphid feeding deterrents and barriers have been reported in the literature, including high secondary metabolite contents in sap (Jakobs et al., 2019), sieve element occlusion (Will et al., 2013), and structural barriers (Kloth et al., 2021). Moreover, aphids are masters of manipulation that not only release effectors that help them to overcome local host defences (Züst & Agrawal, 2016) but also construct niches of their own by changing their host's metabolism (Jakobs et al., 2019). When aphids move between hosts during dispersal, only a small proportion may land on an appropriate host (Ward et al., 2001). Thus, the ability to overcome local feeding barriers may be crucial for the survival of these insects. Several life history traits, including a mixture of viviparous cloning and oviparous sexual reproduction, suggest that dispersal is an Achilles heel for aphids, and switching to xylem feeding could be a parallel behavioral response to sub‐optimal feeding prospects on a particular host. Accordingly, our EPG studies suggest that *C. tremulae* must pass through several phases, with host genotype‐related variations in frequency and duration, to obtain a meal of phloem sap.

Aspens are long‐lived trees with defence traits that substantially vary with variations in both growth environment (Decker et al., 2017) and ontogenetic factors (Cole et al., 2021); aspects that have been largely overlooked to date in evolutionary theories of plant defences (Barton & Boege, 2017; Hahn et al., 2019 and López‐Goldar et al., 2020). Defence response genes in aspen are polymorphic (García & Ingvarsson, 2007; Wang et al., 2016a, 2016b, 2020) suggestively crafted by extended and complex balancing selective processes (Delph & Kelly, 2013; Hurst, 2009), and co‐evolution between aphids and their hosts is indeed likely to be an ongoing process that continuously and simultaneously reshapes plants' defensive traits and mechanisms that enable aphids to cope with those traits (Cope et al., 2021; Züst & Agrawal, 2016). In addition, several piercing‐sucking arthropods live on aspen, including leafhoppers, eriophyid mites, and leaf‐galling aphids of the *Pemphiginae* (Robinson et al., 2012), and resistance might not only protect against piercing damage by one aphid species but against an entire feeding guild as suggested for several plant systems (Kloth et al., 2021; Ng & Perry, 2004; Nombela et al., 2003; von Bargen et al., 2020). Thus, the varied impact of environmental and ontogenetic status on resistance and growth is an expected outcome of balancing selection.

## 5 CONCLUSION

Our results indicate a weak and mostly negative relationship between aphid‐induced CT accumulation and aphid fitness under varied environmental conditions and thus support the general defensive function of CTs that is described in the literature. Our results also show that the resistance of aspen genotypes strongly depends on experimental conditions. Quantitative analyses of the effects variations in ambient temperature, resource availability, and ontogenetic factors have on resistance to aphids could provide further valuable information. In addition, comparative EPG studies of several taxa of piercing‐sucking aspen specialist arthropods may illuminate mechanisms involved in CT‐related feeding behaviors such as xylem sap drinking, and selection strength provided by feeding barriers.

## AUTHOR CONTRIBUTIONS

**Bárbara Díez Rodríguez:** Formal analysis (lead); project administration (lead); validation (lead); visualization (lead); writing—original draft (supporting); writing—review and editing (supporting). **Karen J. Kloth:** Conceptualization (lead); formal analysis (lead); funding acquisition (lead); methodology (lead); software (lead); supervision (supporting); visualization (lead); writing—original draft (supporting); writing—review and editing (supporting). **Benedicte Albrectsen:** Conceptualization (lead); data curation (supporting); formal analysis (supporting); funding acquisition (lead); investigation (lead); methodology (lead); project administration (lead); resources (lead); software (supporting); supervision (lead); validation (supporting); visualization (supporting); writing—original draft (lead); writing—review and editing (lead).

## CONFLICT OF INTEREST

The authors declare no conflicts of interest.

## Supporting information


Figure S1‐S5‐Table S6

## Data Availability

Data supporting findings of this study are openly available in Zenodo at https://doi.org/10.5281/zenodo.5578109.
