# Supplementary material for: Effects of condensed tannins on behavior and performance of a specialist aphid on aspen
Source: Ecol Evol. 2022 Aug 23;12(8):e9229. doi: 10.1002/ece3.9229 (PMC9396707; doi:10.1002/ece3.9229)
Supplement: Supplementary file 1 — Figure S1‐S5‐Table S6 [file ECE3-12-e9229-s001.docx]

## Supporting Information

### Figure S1. Timeline of studies with indicated plant material from the Swedish Aspen collection. Genotypes SwAsp*XX* were classed as genotypes with innately high or low contents of condensed tannins (CTs). Blue and red colours indicate low and high CT levels, respectively. For information about the CT-classification and TanAsp field site see Bandau et al. (2021).


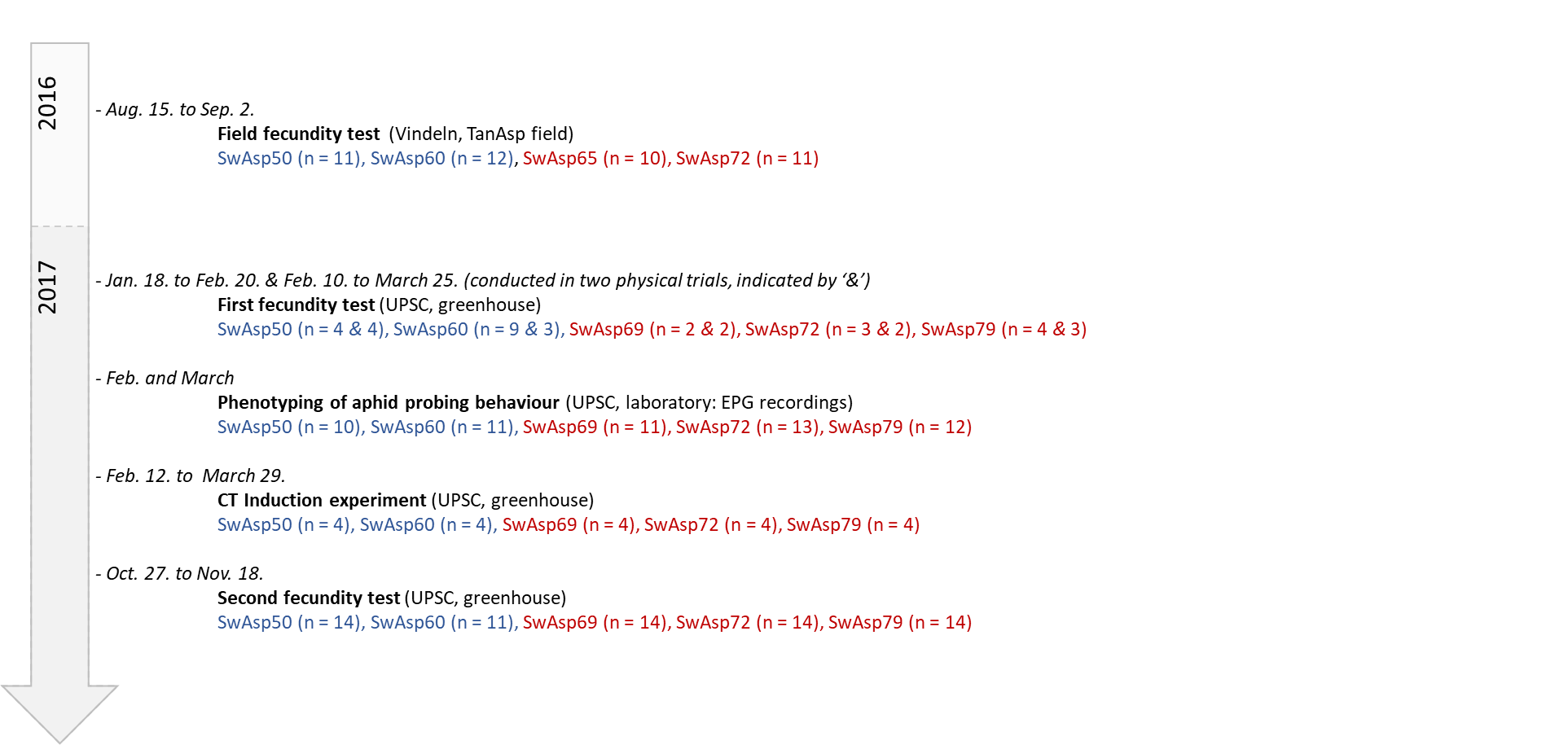


### Figure S2. Images uploaded for determination of *Chaitophorus tremulae* to <https://influentialpoints.com>. A: adult apterous viviparous female. B: nymphs. C: colony with a selection of developmental stages. D: mesh bags used for fecundity studies to hinder aphid escape and exclude enemies. Note the ants that gathered on the mesh bags.


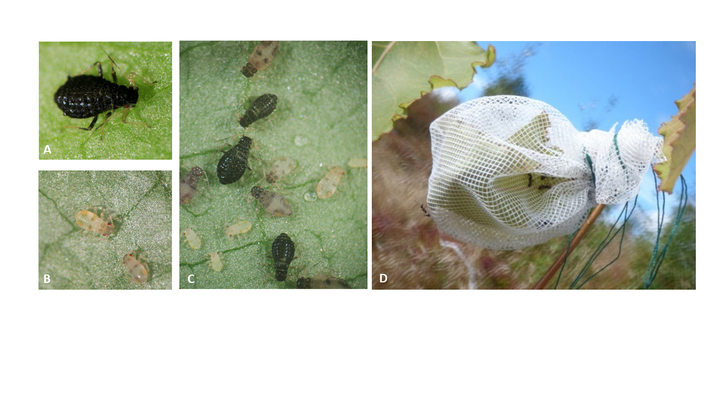


### Figure S3. Overview of the EPG setup, including schematic drawing of the EPG circuit: **A**, illustrative graph obtained with the technique; **B,** illustration of the laboratory setting (**C**) with a close-up of the EPG electrode with a thin gold wire attached to the dorsum of a *C. tremulae* aphid (**D**).


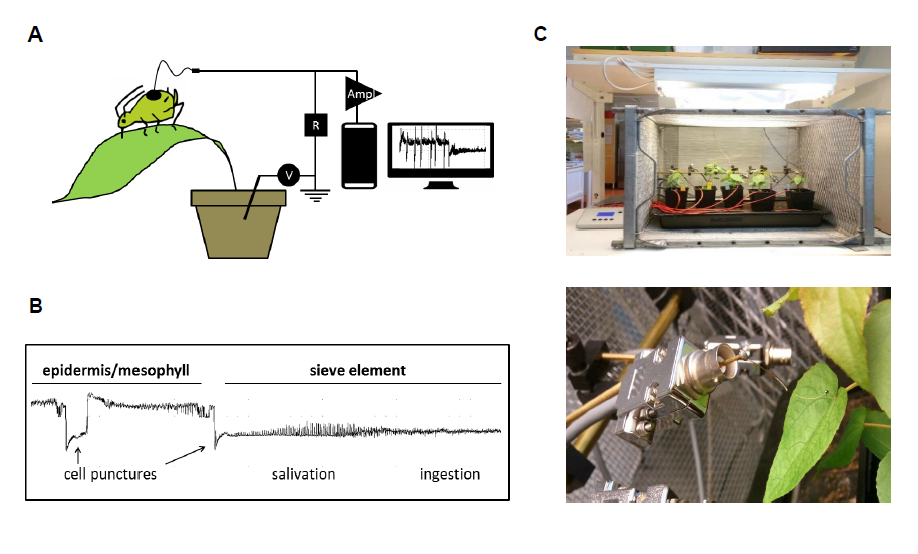


**D**

### Figure S4. Aspen leaves are rich in condensed tannins, with concentrations that vary considerably among genotypes. The images illustrate this variability in leaves of selected genotypes that were included in this study. For localisation studies CTs were stained with 4-dimethylaminocinnamaldehyde (DMACA; blue colour in middle column) following Abeynayake et al. (2011: A high-resolution method for the localization of proanthocyanidins in plant tissues. Plant Methods 7, 13). Images courtesy Dr Fariba Amini.
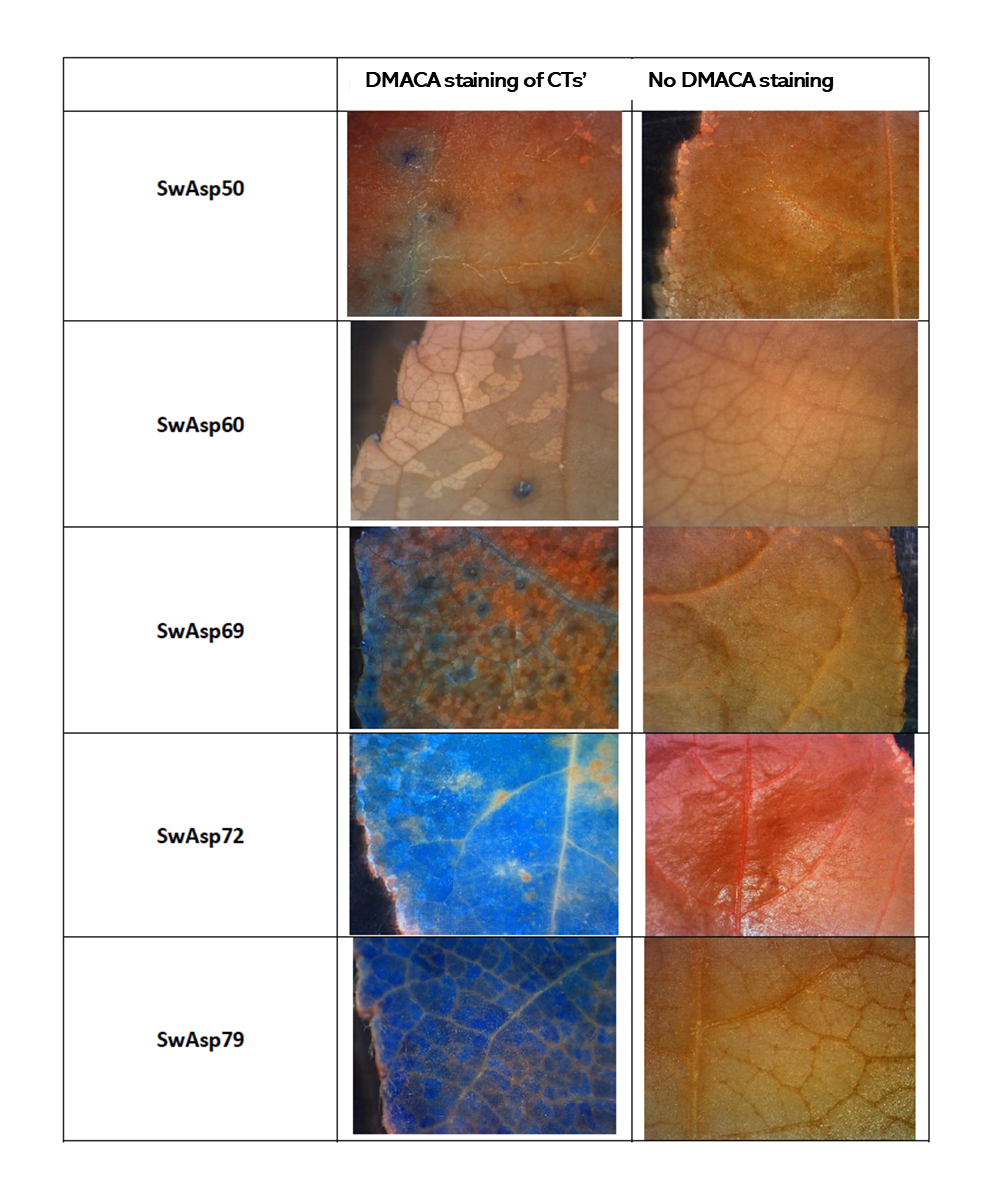


Figure S5. Foliar concentrations of condensed tannins (CT) varied in the tested genotypes (data from greenhouse grown plants). A) CT concentrations measured in constitutive and locally induced leaves. Positive relationships were established between B) aphid numbers and leaf constitutive CT-levels (mg/g DW) measured before infestation and a negative relationship was found between C) aphid numbers and the locally induced CT-level in a leaf after infestation (mg/g DW). The general relationship was supported statistically (Appendix, Table S6). Correlations that considered genotype effects were not significant (two-way-anova with and without interaction term, n= 4 per genotype).


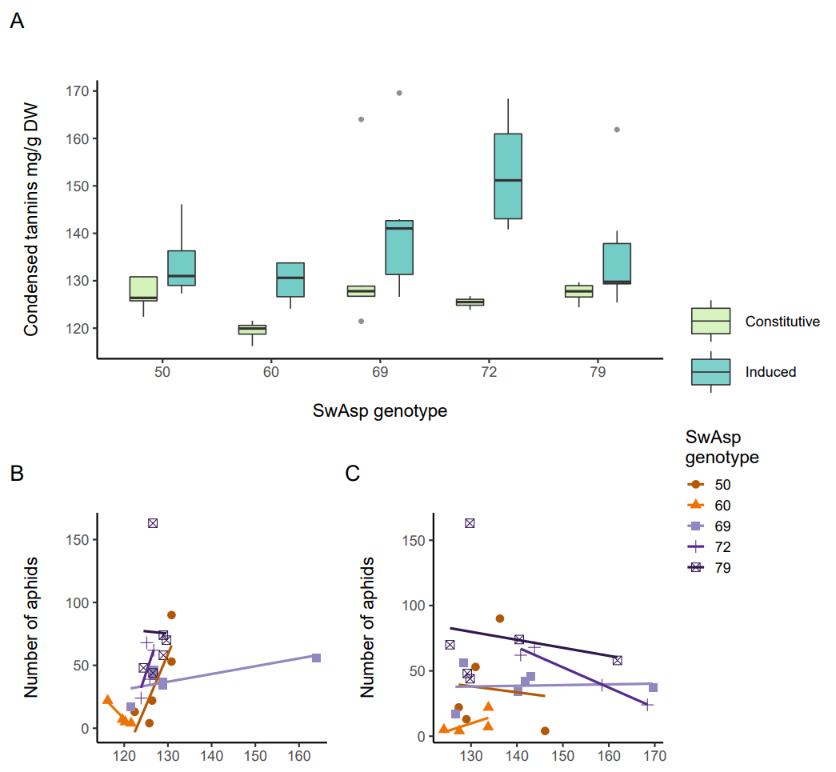


Constitutive CTs before infestation Locally induced CTs after infestation

### Table S6. Correlations between aphid reproduction and foliar condensed tannin concentrations differed between constitutive (positive) and local induced (negative) leaves of aspen plantlets that were grown in the greenhouse. Host genotype affected the total number of aphids (R² conditional) more strongly than foliar tannin concentrations (R² marginal). Correlations were tested with generalized linear models (GLMs) fitted to the Poisson distribution, with tannin concentration as a fixed factor and genotype as a random factor.

|  | Estimate | SE | Z value | Pr(>\|z\|) | R^2^ marginal | R^2^ conditional |
| --- | --- | --- | --- | --- | --- | --- |
| Constitutive | 0.019 | 0.004 | 5.155 | < 0.001 *** | 0.061 | 0.946 |
| Induced | -0.009 | 0.003 | -3.291 | < 0.001 *** | 0.026 | 0.959 |
